# Operations in Cottage Hospitals

**Published:** 1903-07-04

**Authors:** 


### OPERATIONS IN COTTAGE HOSPITALS.

A recent visit to an excellent cottage hospital, which we purposely do not name, situated in one of the most healthy and beautiful parts of the country, showed it to be exceptionally well kept, and to contain every up-to-date and modern appliance, including one of the very best small operation theatres we have ever come across. On examining the in-patient book it turned out that no serious cases for operation had been admitted for 12 months, and the only one coming under this category which had been received was sent to a London hospital for operation. This week we have received a letter asking us whether the following operations ought to be performed at a cottage hospital of eight beds in the course of one year, during which 72 patients were admitted: four abdominal sections, three colotomies, one amputation of thigh, in addition to 24 smaller operations in which are included cases of cancer, strangulated hernia, and tumours? Both sides of the question are here presented in a nutshell. We have always held that the cottage hospital offers great advantages to every class of residents in country districts when the medical staff are efficient, because under capable management it attracts to the country really good men, who are enabled to keep their hands in and themselves up-to-date in matters of treatment. Where these conditions are present the best security is afforded to all residents in the country, including the wealthy as well as the poor. Of course, if the operations mentioned are to be undertaken in a cottage hospital, it is essential that the nurse-matron shall be a first-rate surgical nurse, and that she shall have competent assistance. What the latter may entail must be decided in each cottage hospital, with due regard to the pressure upon the beds and the class of cases admitted from time to time. In every such hospital where serious operations are performed there must be at least two fully-trained and good surgical nurses, of whom one should be the matron. We think the inhabitants of the cottage hospital district where the major operations were successfully performed in one year are to be congratulated on the quality of the medical service which they have succeeded in securing for their protection and safety. We hope they will be willing to supply sufficient money to defray the cost of a second surgical nurse, and to do their part to support the medical staff as they deserve to be supported.

